# Diagnostic performance of a tear protein panel in early dry eye

**Published:** 2013-06-06

**Authors:** Piera Versura, Alberto Bavelloni, Marco Grillini, Michela Fresina, Emilio C. Campos

**Affiliations:** 1 Ophthalmology Unit, DIMES, Alma Mater Studiorum University of Bologna, Bologna, Italy; 2 Laboratory of Musculoskeletal Cell Biology-RAMSES-Rizzoli Orthopedic Institute, Bologna, Italy

## Abstract

**Purpose:**

To evaluate the tear protein pattern in patients with recent subjective symptoms of dry eye (DE) and with poor distinctive DE clinical signs.

**Methods:**

One hundred sixty patients suspected of suffering from mild to moderate DE according to the Dry Eye Workshop (DEWS report 2007) severity grade and 45 matched normal volunteers were included in the study. Subjective symptom score (Ocular Surface Disease index score), Schirmer test I, tear film break-up time, cornea and conjunctiva staining (National Eye Institute score); and tear protein analysis were performed. Statistical evaluation of data was performed with Mann–Whitney unpaired and Student *t* tests, (significance p<0.05). Correlations between variables were evaluated by using Pearson's (r) or Spearman's (ρ) correlation coefficients. Thresholds were selected from receiver operating curves; sensitivity, specificity, likelihood ratio (LR+), and positive predictive values were calculated for each protein. The combination of variables was carried out by univariate analysis, representing the best combination of tests for early DE diagnosis.

**Results:**

Total protein content (TP) and the following proteins were recognized in all samples: lysozyme-C (LYS-C), lactoferrin (LACTO), tear lipocalin 1 (LIPOC-1), zinc-alpha-2-glycoprotein (ZAG-2), transferrin (TRANSF), and exudated serum albumin (ALB). A statistically significant decrease was demonstrated between normal subjects and patients with DE (mg/ml, mean±SD) for TP (9.89±2.28 versus 6.44±2.1), LYS-C (3.06±1.07 versus 2.15±0.78), LIPOC-1 (1.71±0.52 versus 0.98±0.5), ZAG-2 (0.43±0.24 versus 0.25±0.2), TRANSF (0.9±0.6 versus 0.33±0.3), and LACTO (2.11±0.74 versus 1.47±0.76), while an increase was found for ALB (0.21±0.5 versus 0.94±1.28). LIPOC-1 and ZAG-2 were strongly correlated to tear film break-up time. The proteins were related to the DEWS severity grade. Changes in each protein were a better predictor of early DE than were clinical variables; TP, LIPOC-1, and ALB exhibited the highest diagnostic performance either alone (LR+ 16.7, 12.3, 4.7, respectively) or when combined in a univariate analysis (LR+: 41.8, positive predictive value: 99.9).

**Conclusions:**

Our results demonstrated in tears from patients with early DE a significant reduction in tear protein content as a whole, associated with a decrease in proteins with antibacterial and protective functions. A decrease in proteins with lipid binding properties and an increase in inflammatory-related proteins were also shown. Changes in the abundance of a panel of tear proteins with divergent functions was found to better diagnose early DE than did conventional clinical tests.

## Introduction

Dry eye (DE) is recognized as a multifactorial chronic disease, evolving gradually through variable severity stages as homeostatic mechanisms fail to compensate in response to multiple stress factors over time [1]. A weak correlation exists between subjective symptoms of discomfort and objective clinical parameters. This is particularly true in the early stages when clinical signs may be mild or lacking all together [2].

Increasing scientific evidence supports tear protein analysis as a promising candidate for broadening the knowledge surrounding the pathology of DE as changes in tear proteomes can reflect the state of the ocular surface and highlight disease state. The human tear protein pattern has been evaluated in the past with several analytical methods [3], with particular reference to changes in advanced stages of DE as occurs in patients with Sjögren’s syndrome [4-7]. The most recent mass spectrometry research has been dedicated to discovering protein biomarker(s) that could assist with early diagnosis or follow-in DE progression [8-11]; however, integration of human tear proteomics into daily clinical activity is still in progress. Analysis of tear film protein composition in routine practice has been hampered in the past primarily by poor sample size and variability in collection methods. Therefore, determining a protein candidate is a diagnostic marker in the everyday routine is still an unmet need.

In previous works [12,13], the application of a chip-based miniaturized capillary gel electrophoresis device in evaluating human tear proteins was demonstrated. Our group, in particular, validated the system [13] and the quantitative evaluation of the tear protein pattern, which could have been of potential interest in DE early diagnosis. The purpose of the present work was to analyze tears from normal subjects and from patients with early to mild DE and to calculate the diagnostic performance of these proteins either alone or grouped in a panel.

## Methods

A total of 205 subjects, including 160 patients clinically diagnosed as suffering mild to moderate DE and 45 healthy controls, were included; details about the study population are given in Table 1. The study was conducted in accordance with the current ethical principles of the Declaration of Helsinki. The visits were all carried out during morning office hours, approximately 8 AM through 1 PM.

**Table 1:** Demography of the population included in the study according to age (medium ±SD) and gender.

**number**	**controls**	**DE patients**	**total**
males	17	24	41
females	28	136	164
total/subgroup	**45**	**160**	**205**
**age (yrs)**	**controls**	**DE patients**	**total**
males	45.5±11.2	49.3±19	47.6±16.1
females	43.2±8.4	51.4±15.9	49.9±15.2

Patients were classified as Grade 1–2 DE severity according to a modified Dry Eye Workshop (DEWS) scheme (Table 2) [2,14]. Patient inclusion criteria were as follows: mild subjective symptoms of ocular discomfort (for at least 3 months but not more than 1 year) as evaluated with an Ocular Surface Disease index (OSDI) questionnaire [15] (score 12–30) and occasional use of tear substitutes, suspended since at least 1 day before tears were collected.

**Table 2:** DE severity grade according to DEWS guidelines modified by Asbell and Lemp [11].

**Variables**	**1**	**Severity**	**grade**	**4**
		**2**	**3**	
Symptoms-OSDI score	12–15	16–30	31–45	>45
TFBUT, sec	8–15	<10	<5	immediate
Schirmer test, mm /5 min	<10–15	<10	<5	<2
Corneal staining, NEI scale 0–15	0–3	1–8	9–14	14–15
Conjunctival staining, NEI scale, 0–18	0–3	1–7	8–14	15–18
**Number of patients**	**55**	**105**		

Description of the severity grades is as follows: mild dry eye disease (DEWS severity 1 and 2), moderate-severe dry eye disease (DEWS severity 3 and 4). Clinicians classified patients of the present study as grade 1 (n=55) or grade 2 (n=105).

Inclusion criteria for healthy control subjects was absence of subjective symptoms of ocular discomfort (OSDI score <12). In both groups, the exclusion criteria considered the presence of autoimmune disease, the use of contact lens, and any ocular surgery in the previous year.

Tear film break-up time (TFBUT) and Schirmer test I were carried out according to DEWS guidelines [16]. Corneal and conjunctival vital staining was performed by using sodium fluorescein and graded according to the National Eye Institute (NEI) grading system [17]. Briefly, corneal staining was graded with a score of 0–3 (0=normal and 3=severe) assigned to each of five corneal zones (superior, nasal, central, inferior, temporal) with a maximum total score of 15. Conjunctival staining was recorded for three areas of temporal and nasal conjunctiva of each eye and graded 0–3 as above for each zone with a maximum score of 18.

Patients were requested to position their head laterally for a minimum of 1 min for tears to accumulate at the outer canthus. A minimum amount (5 µL) of tears was carefully aspirated by using a micropipette with sterile tips, paying attention not to touch the conjunctiva to avoid reflex lachrymation and consequently sample dilution. Aspirated tears were then centrifuged at 13.200 ×g for 15 min, and the supernatant was aspirated and stored in low protein adsorption surface plastic vials at 4 °C until analysis, performed within 2 days. On occasion, the storage period was forcibly prolonged up to 2 weeks, in which case the samples were stored at –20 °C. Chip-based analysis was performed with the Agilent 2100 Bioanalyzer system (Agilent, Waldbronn, Germany), using the LabChip Kit Protein 230, according to the manufacturer’s instructions, as previously described [13]. All reagents were provided with the kit, including the standard protein ladder containing different proteins with known concentration and molecular weights that can be used for semiquantitative analysis. Briefly, 2 μl of tears were diluted in sample buffer with 1 M dithiothreitol solution (DTT), denaturated in boiling water for 5 min, cooled down on ice, and centrifuged for 15 s. Then 84 μl deionized water was added to the ladder and samples. A 6 μl aliquot of this solution was loaded onto the chip, which was first filled with a gel/dye mix and destaining solution. Separated proteins were detected with laser-induced fluorescence. The sample buffer included an upper and a lower marker of known molecular weight.

Total protein (TP) content and peaks for lysozyme C (LYS-C), tear lipocalin-1 (LIPOC-1), zinc-alpha-2-glycoprotein (ZAG-2), serotransferrin (TRANSF), lactotransferrin (LACTO), and exudated serum albumin (ALB) were recognized at given molecular weights (Table 3). Proteins were expressed either as % versus total protein content and as mg/ml tear sample as described earlier [13].

**Table 3:** Summary of the recognized proteins, protein name in Swiss–Prot database, alignment in the virtual Bioanalyzer electropherogram according to kDa range, function.

**Protein name** **(abbreviation)**	**PROT NAME DATABASE**	**kDa range** **Lab-chip Kit 230**	**Function**
Lysozyme (LYS-C)	LYSC_HUMAN (P61626)	14.3 – 15.0	Antibacterial enzyme. Innate immunity
Lipocalin-1 (LIPOC-1)	LCN1_HUMAN (P31025)	18.1 – 19.9	Ability to bind and transport small, hydrophobic molecules
Zinc-α2-glycoprotein (ZAG-2)	ZA2G_HUMAN (P25311)	30.5 – 36.0	Lipid breakdown in adipocytes, specific role in tears not known . Possible role in immunity
Albumin (ALB)	ALBU_HUMAN (P02768)	59.1 – 65.4	Transportation of free fatty acids, stabilizing the osmotic pressure
Lactotransferrin (LACTO)	TRFL_HUMAN (P02788)	93.7 – 99.3	Inhibitor of bacterial growth. Possible anti-inflammatory properties. Innate immunity
		105.2 – 110*	
Serotransferrin (TRANSF)	TRFE_HUMAN (P02787)	73.5 – 78.4	Iron binding transport

* - A shoulder peak for lactotransferrin is found in this analysis performed with the Protein 230 kit

### Statistical analysis

Statistical evaluation was performed using MedCalc and SPSS 14 software, applying the Mann–Whitney and unpaired Student *t* tests. The linear relationship between each protein and each clinical variable was evaluated with Pearson's (r) or Spearman's (ρ) correlation coefficient (range −1 to 1). Strength of correlation was estimated according to coefficient magnitude as follows: 0.5−1.0, high correlation; 0.5–0.3, moderate correlation; and 0.3–0.1, small if any (linear) correlation [18]. Correlations were considered statistically significant at p<0.05.

Diagnostic value for each protein was analyzed for sensitivity (the ability to correctly identify patients with the disease), specificity (the ability to correctly identify patients without the disease), likelihood ratio (LR+, how much more likely is it that a patient who tests positive has the disease compared with one who tests negative), and positive predictive values (PPV, how likely is it that a patient has the disease given that the test result is positive). Parameters for receiver operator characteristic (ROC) curves were calculated and in particular the area under the curve, which represents the overall accuracy of a test, with a value approaching 1.0, indicating high sensitivity and specificity. ROC curves are used to determine a cut-off value for a given clinical test able to classify cases as positive or negative.

The prevalence of DE was calculated having as a reference the population included in the present study. In a univariate analysis, cut-off points from the ROC curves of the proteins and tests were selected and combined into a definition for a positive DE diagnosis, and diagnostic parameters were calculated for any combination panel. Values for p less than 0.05 were regarded as statistically significant.

## Results

**Figure 1:**
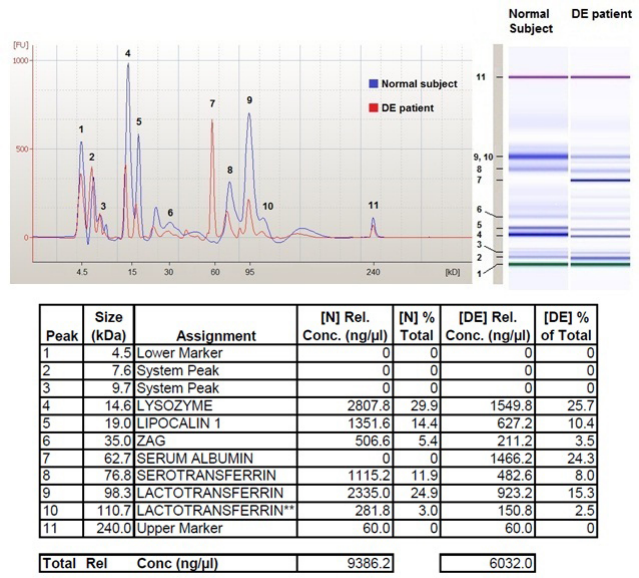
Data from 2100 Bioanalyzer analysis.Upper left: Electropherograms from a representative normal subject (blue line) and a dry eye (DE) patient (red line) are here aligned and overlapped. Recognized peaks of interest are numbered 1 through 11. Upper right: The virtual gel images related to both samples are here compared showing different intensity of corresponding bands between normal subject and DE patient. Bands are here also numbered 1 through 11. Table at the bottom summarizes for each peak the following parameters: recognized molecular weight in kDa, name of the protein assigned on the basis of the validation process [13], concentration of each protein expressed in ng/microliter, percentage of each protein versus total protein content for both (N) normal subject and (DE) patient. The last line of this table reports total protein concentration expressed in ng/microliter

Figure 1 shows overlapping electropherograms from a representative normal subject and a patient with DE with peaks of interest numbered, and the relative virtual gel images. A summary of the recognized proteins and their alignment in the virtual bioanalyzer electropherogram according to kDa range is given in Table 3.

Results from clinical tests are reported in Table 4; the OSDI results had an incorporation bias. The other clinical tests showed statistically significant changes in patients with DE versus controls, except the Schirmer test I, which remained unchanged. The epithelial damage evaluated as the vital staining NEI score, however, was mild in the DE group.

**Table 4:** Descriptive statistics for clinical tests analyzed in normal subjects versus DE patients.

test	value	Normal subjects	DE patients	p
OSDI *	score	3.5±2.1	25.2±4.9	<0.0001
TFBUT	seconds	12.6±1.9	7.8±3.6	<0.0001
Schirmer test I	mm/5 min	24.8±6.2	23±12.1	0.01
Cornea NEI	score	0.06±0.25	1.17±0.96	<0.0001
Conjunctiva NEI	score	0.35±0.41	2.43±1.46	<0.0001

Results are summarized (mean ± SD) for subjective symptom (OSDI, Ocular Surface Disease Index score), Tear Film Break-Up time (TFBUT), Schirmer test I, Corneal and Conjunctival vital staining scored by the National Eye Institute (NEI) grading system. Results from all tests, except Schirmer test 1, were shown to be statistically significant. * incorporation bias

All tear samples were successfully collected and analyzed with the 2100 Bioanalyzer. A 2 μl tear sample was a suitable amount for analysis in the detection system to obtain a well-defined protein separation and a readable virtual band image, without any overlapping of the bands of interest.

The recognized protein bands were visible in all samples, but the tear protein profiles were different between the normal subjects and the patients with DE. In particular, a statistically significant decrease was demonstrated between the normal subjects and the patients with DE (mean±SD) for TP, LIPOC-1, ZAG-2, TRANSF, and LACTO, while an increase was observed for ALB. Data are summarized as relative % versus total tear protein and as absolute content in mg/ml tear (Table 5).

**Table 5:** Summary of the results for normal control subjects and DE patients (medium + SD), data are expressed as percentage (%) versus total content and mg/ml sample.

**Analite**	**% versus total protein content**		**p**	**mg/ml**		**p**
	**Normal subjects**	**DE patients**		**Normal subjects**	**DE patients**	
LYS-C	34.96±6.78	34.92±9.49	0.09	3.06±1.08	2.15±0.78	<0.0001
LACTO	24.14±5.08	21.44±6.69	0.01	2.11±0.74	1.4±0.76	<0.0001
LIPOC-1	18.26±4	15.27±6.03	0.0002	1.71±0.52	0.98±0.49	<0.0001
TRANSF	9.58±2.96	4.52±3.78	<0.0001	0.89±0.67	0.33±0.36	<0.0001
ALB	4.27±4	11.91±12.82	<0.0001	0.21±0.55	0.94±1.28	<0.0001
ZAG-2	4.94±1.05	3.13±2.28	<0.0001	0.43±0.24	0.25±0.22	<0.0001
Total Protein (TP)				9.89±2.28	6.44±2.1	<0.0001

Total Protein content is only expressed in absolute value mg/ml. A statistically significant decrease was demonstrated in all proteins, except albumin that showed a significant increase in DE patients versus controls.

All proteins except LYS-C showed the same trend either as relative or absolute content. The relative % of LYS-C did not significantly change in tears from patients with DE patients versus tears from controls.

The correlation values between each protein and clinical variables are summarized in Table 5. Moderate to high correlations were found and in particular between some selected proteins (LIPOC-1, ZAG-2 LACTO, TP) and TFBUT. Low to insignificant correlations between any protein and the Schirmer test I were found. All proteins were inversely correlated to the DEWS severity score, except ALB, for which a direct correlation was found. The OSDI subjective symptom score appeared inversely correlated to TP and LACTO (Table 6).

**Table 6:** Pearson’s r or Spearman’s ρ correlation coefficients between tear protein and clinical parameter (significance p<0.05).

**Test variable**	**OSDI**	**TFBUT**	**Schirmer test 1**	**Cornea** **NEI**	**Conjunctiva NEI**	**DE severity** **score**
**LYS-C**	r=- 0.28 p=0.0001	r=0.11 p=0.12	r=0.12 p=0.12	r=-0.28 p=0.0001	r=-0.16 p=0.01	ρ=- 0.39 p<0.0001
**LACTO**	r=-0.35 p<0.0001	r=0.35 p<0.0001	r=0.07 p=0.2	r=-0.30 p<0.0001	r=-0.24 p=0.0006	ρ=- 0.36 p<0.0001
**LIPOC-1**	r=- 0.31 p<0.0001	r=0.39 p<0.0001	r=0.16 p=0.02	r=- 0.42 p<0.0001	r=-0.23 p=0.001	ρ=- 0.57 p<0.0001
**TRANSF**	r=-0.28 p<0.0001	r=0.17 p=0.01	r=-0.20 p=0.003	r=-0.30 p<0.0001	r=-0.24 p=0.0006	ρ=-0.37 p<0.0001
**ALB**	r=0.09 p=0.1	r=- 0.22 p=0.001	r=-0.15 p=0.4	r=0.16 p=0.02	r=0.21 p=0.002	ρ=0.52 p<0.0001
**ZAG-2**	r=-0.16 p=0.03	r=0.41 p<0.0001	r=0.13 p=0.7	r=-0.3 p=0.001	r=-0.12 p=0.2	ρ=-0.35 p<0.0001
**TP**	r=-0.35 p<0.0001	r=0.33 p<0.0001	r=-0.06 p=0.4	r=- 0.36 p<0.0001	r=-0.33 p=0.001	ρ=- 0.54 p<0.0001

OSDI, ocular surface disease index; TFBUT, tear film break-up time, Corneal and Conjunctival vital staining scored by the National Eye Institute (NEI) grade system. DE severity was scored as 1 or 2 according to Asbell and Lemp [14].

The diagnostic accuracy for each protein was assessed and compared, and a cut-off value assigned for each protein. In Table 7, the results from the ROC curve analysis in patients with mild to moderate DE versus controls are listed. As a test for DE, the cut-off points were selected with emphasis on specificity to compensate for the loss of specificity that inevitably occurs when variables are combined as a test for DE. Clinical test performance suffered to some extent by bias derived from predefined inclusion criteria; despite this, the Schirmer test I showed poor diagnostic performance. High specificity and PPVs were found for all proteins.

**Table 7:** Diagnostic performance of clinical tests and proteins.

Test variable	Cut-off points	Specificity	Sensitivity	LR+	PPV	ROC curve	analysis	parameters
						Area under the curve	Standard error	95% confidence interval
Schirmer test I	≤10 mm / 5′	84.4	37.7	2.43	89.6	0.594	0.049	0.523 – 0.662
TFBUT	≤10 s	76.6	75.5	3.13	91.5	0.87	0.035	0.816 – 0.914
Cornea NEI	score >0	93.3	30.6	5,04	21,0	0,664	0,054	0,566 – 0,754
Conjunctiva NEI	Score >0	66,7	68.7	10.3	97.3	0.824	0.03	0.765 – 0.874
ZAG-2	≤0.4 mg / ml	87.5	56.9	2.03	81.8	0.759	0.041	0.691 – 0.819
ALB	>10%versus total TP	90.9	42.5	4.7	97.1	0.729	0.05	0.657 – 0.793
LACTO	≤1.2 mg / ml	93.2	44.2	6.4	94.5	0.812	0.036	0.753 – 0.861
LIPOC-1	≤1.1 mg / ml	94.9	65.5	12.3	97.1	0.843	0.034	0.787 – 0.888
TP	≤6.5 mg / ml	96.6	55	16.7	97.8	0.861	0.032	0.808 – 0.904
LYS-C	≤2.0 mg / ml	96.6	57.8	17.7	97.9	0.826	0.032	0.813 – 0.908
TRANSF	≤0.3 mg / ml	96.7	70.8	18	98.1	0.885	0,030	0.798– 0.903

Specificity, sensitivity, positive likelihood ratio (LR+) Positive Predictive Value (PPV+) and parameters of ROC curve analysis for each test or protein were calculated comparing normal control subjects versus DE patients. Data are listed on increasing value of LR+. Explanation in the text, sections Methods and Discussion

To improve the diagnostic potential of the tests, different combinations of variables were associated, and their performance was calculated; the most significant combinations are summarized in Table 8. The combination of TFBUT, Schirmer Test I, and corneal vital staining NEI score exhibited high PPVs (Table 8 combination #1 and #2), but this was possibly related to an inclusion bias error. Combinations of different proteins showed the highest performance: the association TRANSF - TP - LIPOC-1 (Table 8, combination #3) showed the highest specificity and sensitivity values, while the association ALB - TP - LIPOC-1 (Table 8, combination #4) reached a PPV of 99.9 and a 20-fold increase in the LR+ value compared to the standard association of TFBUT and Schirmer test I.

**Table 8:** Clinical tests and tear proteins were combined as a test for early DE diagnosis.

**Combination** **n.**	**Criteria for classifying** **DE variable (cut off point)**	**Specificity**	**Sensitivity**	**LR+**	**PPV**
**1**	TFBUT ≤10 s OR Schirmer test I ≤10 mm / 5′	60.0	84.0	2.11	88.0
**2**	TFBUT ≤10 s OR Cornea NEI score >0	90.0	93.0	13.4	96.1
**3**	TRANSF ≤0.3 mg/ml OR TP ≤6.5 mg / ml OR LIPOC-1 ≤1.1 mg / ml	96.0	98.0	20.9	99.0
**4**	ALB >10%versus total TP OR TP ≤6.5 mg / ml OR LIPOC-1 ≤1.1 mg / ml	98.0	91.0	41.8	99.9

The table summarizes the combinations with the best diagnostic performance. The combination of TFBUT, Schirmer Test I, corneal vital staining NEI score exhibited high PPV values (combination #1 and #2). The association TRANSF - TP - LIPOC-1 (combination #3) showed the highest values of specificity and sensitivity, while the association ALB - TP - LIPOC-1 (combination #4) reached a PPV 99.9 value and a 20 fold LR+ value as compared to association of clinical tests.

## Discussion

DE is an often underestimated condition, but increasing scientific evidence over the last few years has highlighted its high prevalence, and DE is now considered a significant healthcare problem. DE impairs not only patients' quality of life but also impacts the economy in many aspects [19]; thus, early diagnosis and effective appropriate therapy are now addressed issues.

Tear proteomics is a promising area of research exploring the specific protein complexes able to differentiate patients with DE from control subjects as well as new technologies to objectively confirm patients’ subjective discomfort. Early mass spectrometry–based studies missed many of the changes in proteins present due to the inherent design of the procedures that foresee the analysis of equal amounts of total protein in the absence of variables for volume or quantitation. Thus, these analyses simply demonstrated which proteins were present. A recent study using quantitative mass spec (iTRAQ) demonstrated equivalent variations in the proteins we present here [11]. Unfortunately, iTRAQ analysis is not a clinical option for analyzing individual patients for many obvious reasons. Some biomarkers have been recently proposed [20], such as interleukin-1 receptor antagonist (IL-1Ra), interleukin-8 [9], and other tear cytokines, chemokines, and soluble receptors [8,21] shown to be associated with DE clinical signs and disease severity. Enhanced matrix metalloproteinase-9 was also demonstrated to be a biomarker for diagnosing inflammation in DE [10]. In contrast, lachrymal proline-rich 4 protein in tears specifically from lachrymal gland secretion were significantly downregulated in all types of dry eye, correlating with disease severity [22]. Lacritin also appears to be downregulated in DE [23], and this finding may explain impairment of autocrine/paracrine functions of the lachrymal function unit such as basal secretion. Tear osmolarity measurement has also been proposed as a biomarker of dry eye severity [24] with high diagnostic performance [25].

Previously, the 2100 Bioanalyzer was shown to provide total tear protein content and concentration of specific tear proteins with significant time saving, improved ease of use, and high reproducibility compared to traditional one-dimensional sodium dodecyl sulfate–polyacrylamide gel electrophoresis [12,13]. The use of this “lab-on-a-chip” device together with the Protein 230 LabChip kit was validated and its use proposed for the routine clinical practice to quantitatively estimate the concentration of several protein species [13].

The present study was performed in patients with mild DE at an early stage, when diagnosis is still problematic as clinical signs are often poor or lacking at all. Data were obtained by analyzing samples from single subjects, and pooling was not necessary, unlike how it is often reported in proteomic studies. As documented with the normal Schirmer test I values recorded in patients with DE included in the study, a suitable amount of tears was always detectable, which made collecting the tears needed for analysis easier. In normal subjects, the amount of total tear protein, the profile of major proteins, and their individual percentage agreed with previous literature [26]. Our data also showed that in patients with early DE a reduction in tear protein content as a whole was associated with a decrease in proteins that may have protective functions and lipid binding properties, and an increase in inflammatory-related proteins.

In the previous literature, changes in the tear protein profile were recognized in severe DE as in patients with Sjögren’s syndrome [6,27]. As stated, DE is a progressive disease, with phases of severity in waves [1]. Our results demonstrate that protein changes occur at an early DE stage and may suggest the role of these changes in the failure of homeostasis from the beginning.

Analysis of total protein content in a given biologic fluid sample is a basic method that provides information on general health conditions; abnormal total protein levels indicate further tests are required to investigate major organ involvement. In this study, the total proteins present in tears from patients with DE were decreased compared to normal unaffected control subjects, suggesting an impairment of lachrymal function unit synthesis capabilities.

Lachrymal gland–derived antimicrobial tear proteins, lysozyme and lactoferrin, showed slightly different results in this study, but the trend was strongly related. The percentage versus total protein content value remained unaltered for lysozyme while a limited decrease was shown for lactoferrin in the tears from patients with DE versus the tears from controls. Both proteins, in fact, were the two most abundant species in the control and DE tear profiles. On the contrary, the absolute content of lysozyme and lactoferrin significantly decreased in tears from patients with DE versus control tears, demonstrating a decrease in lachrymal gland synthesis, as they represent gland function [28]. In addition to other mechanisms, a decrease in lactoferrin can contribute to an increase in inflammatory markers in tears, as has been suggested in keratoconus [29].

Tear lipocalins, representing the largest group of lipid affinity proteins in tears, are produced in the lachrymal gland as well as in von Ebner’s gland and can bind to a wide variety of lipids favoring lipid solubility in the tear film during blinking [30]. Lipocalin decreases in tears from patients with meibomian gland dysfunction [31] and patients with early stage hyperevaporative DE [32] were demonstrated in past literature. In the present study, lipocalin-1 remained the third most abundant species in the profile, but its content was reduced in DE versus control tears. Lipocalin-1 also showed an inverse correlation versus TFBUT, thus confirming what was previously demonstrated [32] by our group, strongly supporting the role of this protein in tear stability mechanisms. Taken as a whole, the findings were consistent with an initial impairment of the lachrymal gland at this early DE stage.

Zinc-alpha-2-glycoprotein, a 41-kDa protein, is a common component of many body fluids including tears, which is [33] secreted by various normal epithelia. Zinc-alpha-2-glycoprotein has been shown to stimulate lipolysis in adipocytes and to be associated with the extreme weight loss that occurs in some cancers [33,34]. However, the specific role of ZAG-2 in tears is not known. Due to its structural similarity to the major histocompatibility complex class I antigen-presenting molecule, a role in the immune response has been hypothesized [35]. We demonstrated a decrease in ZAG-2 in this study, in agreement with previous work [29,32]. Of course, correlation does not imply causation, but a direct correlation between ZAG-2 and TFBUT was found, which may support a role in managing lipids in tear secretions; further studies are needed in this regard.

Plasma-derived major proteins, serum albumin and transferrin, behaved differently in tears from patients with DE versus tears from controls. Both proteins derive from plasma through a passive filtration from vessels, but in normal tears, serum albumin content is extremely low (nearly negligible) while transferrin represents the fourth most abundant species, about 10% of the total protein content [26]. Transferrin is an iron-binding monomeric glycoprotein belonging to the innate immune system of the eye. Along with lactoferrin, transferrin contributes to the iron-sequestration mechanisms active against pathogens [36]. In the present study, a significant increase in albumin and a significant decrease in transferrin were shown in tears of patients with early DE versus controls.

Serum albumin is an indirect sign of subclinical inflammation as it occurs when there is an increase in protein leakage from inflamed conjunctival vessels [12,37]. This finding is in agreement with other analyses of the tear proteome [32,38]. In the present study, the serum albumin increase in tears occurred in the absence of any clinical sign suggesting inflammation or detectable conjunctival lesions. However, increased rates of evaporation could also contribute to elevated serum albumin concentrations, even if the initial tear composition is normal. DE is recognized as a “multifactorial disease… accompanied by increased osmolarity of the tear and inflammation of the ocular surface” [39]. Our findings suggest that albumin dosage may be a useful marker in objectively assessing ocular surface inflammatory status when clinical signs of disease are not evident.

Transferrin is an iron-binding plasma glycoprotein with a molecular mass of 78 kDa, found in many body fluids and primarily secreted by the liver but also by epithelia, including eye tissues [40]. Lachrymal gland epithelial cells synthetize transferrin at least in in vitro models [41]; transferrin is transported through transcytotic pathways into lachrymal fluid by lachrymal acini in vivo [42]. Two transferrin isoforms, β1 and β2, can be detected in tears (as well as in ear secretions, saliva, and cerebral serum fluid) and their relative variation is an early marker indicating cerebral serum fluid leakage in central nervous system pathology [43]. We did not detect the two isoforms reported to be dissociated by using β-mercaptoethanol, since a sample buffer without this reducing agent is recommended in the Bioanalyzer system. As reported, transferrin can potentially derive from plasma and epithelia, but our analysis did not distinguish the origin. The main outcome, however, is that a decrease in transferrin results in an iron imbalance in tears and consequently leads to cell damage associated with unbound iron. Low levels of transferrin are now recognized as useful markers of inflammation and disease activity and correlate well with inflammatory cytokine levels in autoimmune disease [44].

One intriguing issue is why plasma-derived serum albumin and transferrin behave differently in tears of patients with DE. The present data may explain only the observation of increased albumin leakage from inflamed conjunctival vessels and decreased transferrin synthesis and secretion by the lachrymal gland. The opposite variations of these proteins in tears cannot be explained by a passive filtration mechanism only, and we argue that some unknown controlling factors may take place in the passage through the blood–epithelial barrier.

The demonstrated variations in the proteins presented in this study were not produced by lachrymal stimulation during sampling, as we carefully aspirated tears atraumatically. In addition, stimulated samples were shown to have a decreased, not an increased, level of albumin; further, no substantial change in lactoferrin, tear-specific prealbumin, or lysozyme occurred during overstimulation [45].

In this study, we analyzed the potential of tear proteins as markers for diagnosing patients with early DE, compared with the most common clinical tests used in daily practice. Many papers in the literature demonstrated the low performance of the Schirmer test I, especially in the early stage of DE disease, and suggested a lower cut-off level to gain accuracy [16]. However, when the disease is at the onset, Schirmer test I may not show any loss of gland function: whereas, the protein composition may already have changed significantly, as we demonstrated in the present work. Discussion in detail of these aspects is beyond the scope of this paper; we refer the reader to a comprehensive review of the issue by the International Dry Eye Workshop [16].

The diagnostic performance of all the proteins taken separately was higher than the values exhibited by any clinical test to date, with particular reference to values demonstrated by total protein content, albumin, and lipocalin-1. These three parameters also showed the highest correlation coefficient with an estimated DE severity score 1 or 2 [13].

Due to DE natural history, a combination of variables rather than a single one likely performs better as a diagnostic test to cover all stages of the disease. Thus, we addressed the question whether combined testing yields meaningful gains in performance compared with using one test alone. Several options were estimated, and the overall enhanced performance of protein combinations versus clinical test combinations was demonstrated. This is true in particular for the likelihood ratio (LR) value, an index that incorporates the sensitivity and specificity of the test and gives the estimation of how much a test result will change the odds of having the disease. High LR (e.g., LR>10) indicate that the test can be used to rule in the disease. Our data demonstrated that the combination of Schirmer test I and TFBUT increases the odds of having the disease only by 2, while the combination of albumin, total protein content, and lipocalin 1 at the given thresholds increases the odds by 41. In addition, the PPV of this combination of proteins estimates that the value is true with a percentage of 99.9%.

In the present research, we proposed a tear protein panel that can support clinicians in diagnosing mild and early stages of DE. The method can provide validated data for trials investigating clinical/therapeutic outcomes, and is suitable for affordable application in a clinical setting, in terms of time and costs.

## References

[1] Bron AJ, Yokoi N, Gafney E, Tiffany JM (2009). Predicted phenotypes of dry eye: proposed consequences of its natural history.. Ocul Surf.

[2] Nichols KK, Nichols JJ, Mitchell GL (2004). The lack of association between signs and symptoms in patients with dry eye disease.. Cornea.

[3] Zhou L, Beuerman RW (2012). Tear analysis in ocular surface diseases.. Prog Retin Eye Res.

[4] Tomosugi N, Kitagawa K, Takahashi N, Sugai S, Ishikawa I (2005). Diagnostic potential of tear proteomic patterns in Sjogren’s syndrome.. J Proteome Res.

[5] Giusti L, Baldini C, Bazzichi L, Bombardieri S, Lucacchini A (2007). Proteomic diagnosis of Sjögren's syndrome.. Expert Rev Proteomics.

[6] Versura P, Frigato M, Cellini M, Mulè R, Malavolta N, Campos EC (2007). Diagnostic performance of tear function tests in Sjogren's syndrome patients.. Eye (Lond).

[7] Nguyen CQ, Peck AB (2009). Unraveling the pathophysiology of Sjogren syndrome-associated dry eye disease.. Ocul Surf.

[8] Boehm N, Riechardt AI, Wiegand M, Pfeiffer N, Grus FH (2011). Pro-inflammatory cytokine profiling of tears from dry-eye patients by means of antibody microarrays.. Invest Ophthalmol Vis Sci.

[9] Huang JF, Zhang Y, Rittenhouse KD, Pickering EH, McDowell MT (2012). Evaluations of tear protein markers in dry eye disease: repeatability of measurement and correlation with disease.. Invest Ophthalmol Vis Sci.

[10] Sambursky R, Davitt WF, Latkany R, Tauber S, Starr C, Friedberg M, Dirks MS, McDonald M (2013). Sensitivity and specificity of a Point-of-care Matrix Metalloproteinase 9 immunoassay for diagnosing inflammation related to Dry Eye.. JAMA Ophthalmol.

[11] Srinivasan S, Thangavelu M, Zhang L, Green KB, Nichols KK (2012). iTRAQ quantitative proteomics in the analysis of tears in dry eye patients.. Invest Ophthalmol Vis Sci.

[12] Mann AM, Tighe BJ (2007). Tear analysis and lens–tear interactions Part I. Protein fingerprinting with microfluidic technology.. Cont Lens Anterior Eye.

[13] Versura P, Bavelloni A, Blalock W, Fresina M, Campos EC (2012). A rapid standardized quantitative microfluidic system approach for evaluating human tear proteins.. Mol Vis.

[14] Asbell PA, Lemp MA. *Dry Eye Disease:* The Clinician’s guide to diagnosis and treatment. New York: Thieme; 2007.

[15] Schiffman RM, Christanson MD, Jacobsen G (2000). Reliability and validity of the Ocular Surface Disease Index.. Arch Ophthalmol.

[16] (2007). No authors listed. Methodologies to diagnose and monitor Dry Eye disease. In: 2007 Report of the International Dry Eye WorkShop (DEWS).. Ocul Surf.

[17] Lemp MA (1995). Report of the National Eye Institute/Industry workshop on clinical trials in Dry Eyes.. CLAO J.

[18] Bioinformatics FMH. applications in life and environmental sciences. Springer 2009, pp. 110.

[19] Friedman NJ (2010). Impact of dry eye disease and treatment on quality of life.. Curr Opin Ophthalmol.

[20] Enríquez-de-Salamanca A, Bonini S, Calonge M (2012). Molecular and cellular biomarkers in dry eye disease and ocular allergy.. Curr Opin Allergy Clin Immunol.

[21] Na KS, Mok JW, Kim JY, Rho CR, Joo CK (2012). Correlations between tear cytokines, chemokines, and soluble receptors and clinical severity of dry eye disease.. Invest Ophthalmol Vis Sci.

[22] (2012). Aluru SV, Agarwal S, Srinivasan B, Iyer GK, Rajappa SM, Tatu U, Padmanabhan P, Subramanian N, Narayanasamy A. Lacrimal proline rich 4 (LPRR4) protein in the tear fluid is a potential biomarker of dry eye syndrome.. PLoS ONE.

[23] McKown RL, Wang N, Raab RW, Karnati R, Zhang Y (2009). Lacritin and other new proteins of the lacrimal functional unit.. Exp Eye Res.

[24] Sullivan BD, Crews LA, Sönmez B, de la Paz MF, Comert E, Charoenrook V, de Araujo AL, Pepose JS, Berg MS, Kosheleff VP, Lemp MA (2012). Clinical utility of objective tests for dry eye disease: variability over time and implications for clinical trials and disease management.. Cornea.

[25] Versura P, Profazio V, Campos EC (2010). Performance of tear osmolarity compared to previous diagnostic tests for dry eye diseases.. Curr Eye Res.

[26] Berta A (1982). A polyacrylamide-gel electrophoretic study of human tear proteins.. Graefes Arch Clin Exp Ophthalmol.

[27] Caffery B, Joyce E, Boone A, Slomovic A, Simpson T, Jones L, Senchyna M (2008). Tear lipocalin and lysozyme in Sjögren and non-Sjögren dry eye.. Optom Vis Sci.

[28] Ohashi Y, Dogru M, Tsubota K (2006). Laboratory findings in tear fluid analysis.. Clin Chim Acta.

[29] Lema I, Brea D, Rodriguez-Gonzalez R, Diez-Feijoo E, Sobrino T (2010). Proteomic analysis of the tear film in patients with keratoconus.. Mol Vis.

[30] Dartt DA (2011). Tear lipocalin: structure and function.. Ocul Surf.

[31] Yamada M, Mochizuki H, Kawai M, Tsubota K, Bryce TJ (2005). Decreased tear lipocalin concentration in patients with meibomian gland dysfunction.. Br J Ophthalmol.

[32] Versura P, Nanni P, Bavelloni A, Blalock WL, Piazzi M, Roda A, Campos EC (2010). Tear proteomics in evaporative dry eye disease.. Eye (Lond).

[33] Hassan M, Waheed A, Yadav S, Singh TP, Ahmad F (2008). Zinc A2-Glycoprotein: a multidisciplinary protein.. Mol Cancer Res.

[34] Russell ST, Zimmerman TP, Domin BA, Tisdale MJ (2004). Induction of lipolysis in vitro and loss of body fat in vivo by zinc-alpha-(2)-glycoprotein.. Biochim Biophys Acta.

[35] Molloy MP, Bolis S, Herbert BR, Ou K, Tyler MI, van Dyk DD, Willcox MD (1997). Gooley. AA, Williams KL, Morris CA, Walsh BJ. Establishment of the human reflex tear two-dimensional polyacrylamide gel electrophoresis reference map: new proteins of potential diagnostic value.. Electrophoresis.

[36] Ratledge C, Dover LG (2000). Iron metabolism in pathogenic bacteria.. Annu Rev Microbiol.

[37] Versura P, Profazio V, Fresina M, Campos EC (2009). A novel scraping cytology score system (SCSS) grades inflammation in Dry Eye patients.. Curr Eye Res.

[38] Nichols JJ, Green-Church KB (2009). Mass Spectrometry-based proteomic analyses in Contact lens-related Dry Eye.. Cornea.

[39] (2007). No authors listed. The definition and classification of Dry Eye disease. In: 2007 Report of the International Dry Eye WorkShop (DEWS).. Ocul Surf.

[40] García-Castiñeiras S (2010). Iron, the retina and the lens: a focused review.. Exp Eye Res.

[41] Nguyen DH, Beuerman R, Halbert C, Qiangwei MA, Sun G (1999). Characterization of immortalized rabbit lacrimal gland epithelial cells.. In Vitro Cell Dev Biol Anim.

[42] Edman MC, Marchelletta RR, Hamm-Alvarez SS. Lachrymal gland overview. In: Ocular periphery and disorders. Dartt DA et al (editors), 2011, Academic Press, Oxford, UK Publisher. pp 68–74.

[43] Görögh T, Rudolph P, Meyer JE, Werner JA, Lippert BM, Maune S (2005). Separation of beta2-transferrin by denaturing gel electrophoresis to detect cerebrospinal fluid in ear and nasal fluids.. Clin Chem.

[44] Vanarsa K, Ye Y, Han J, Xie C, Mohan C, Wu T (2012). Inflammation associated anemia and ferritin as disease markers in SLE.. Arthritis Res Ther.

[45] Fullard RJ, Tucker DL (1991). Changes in human tear protein levels with progressively increasing stimulus.. Invest Ophthalmol Vis Sci.

